# Fine Scale Spatiotemporal Clustering of Dengue Virus Transmission in Children and *Aedes aegypti* in Rural Thai Villages

**DOI:** 10.1371/journal.pntd.0001730

**Published:** 2012-07-17

**Authors:** In-Kyu Yoon, Arthur Getis, Jared Aldstadt, Alan L. Rothman, Darunee Tannitisupawong, Constantianus J. M. Koenraadt, Thanyalak Fansiri, James W. Jones, Amy C. Morrison, Richard G. Jarman, Ananda Nisalak, Mammen P. Mammen, Suwich Thammapalo, Anon Srikiatkhachorn, Sharone Green, Daniel H. Libraty, Robert V. Gibbons, Timothy Endy, Chusak Pimgate, Thomas W. Scott

**Affiliations:** 1 Department of Virology, Armed Forces Research Institute of Medical Sciences, Bangkok, Thailand; 2 Department of Geography, San Diego State University, San Diego, California, United States of America; 3 Department of Geography, University at Buffalo, Buffalo, New York, United States of America; 4 Institute for Immunology and Informatics, University of Rhode Island, Providence, Rhode Island, United States of America; 5 Laboratory of Entomology, Wageningen University, Wageningen, The Netherlands; 6 Department of Entomology, Armed Forces Research Institute of Medical Sciences, Bangkok, Thailand; 7 Department of Entomology, University of California Davis, Davis, California, United States of America; 8 Bureau of Vector-Borne Disease, Department of Disease Control, Thailand Ministry of Public Health, Nonthaburi, Thailand; 9 Division of Infectious Diseases and Immunology, Department of Medicine, University of Massachusetts Medical School, Worcester, Massachusetts, United States of America; 10 Department of Infectious Diseases, State University of New York at Syracuse, Syracuse, New York, United States of America; 11 Fogarty International Center, National Institutes of Health, Bethesda, Maryland, United States of America; Centers for Disease Control and Prevention, Puerto Rico, United States of America

## Abstract

**Background:**

Based on spatiotemporal clustering of human dengue virus (DENV) infections, transmission is thought to occur at fine spatiotemporal scales by horizontal transfer of virus between humans and mosquito vectors. To define the dimensions of local transmission and quantify the factors that support it, we examined relationships between infected humans and *Aedes aegypti* in Thai villages.

**Methodology/Principal Findings:**

Geographic cluster investigations of 100-meter radius were conducted around DENV-positive and DENV-negative febrile “index” cases (positive and negative clusters, respectively) from a longitudinal cohort study in rural Thailand. Child contacts and *Ae. aegypti* from cluster houses were assessed for DENV infection. Spatiotemporal, demographic, and entomological parameters were evaluated. In positive clusters, the DENV infection rate among child contacts was 35.3% in index houses, 29.9% in houses within 20 meters, and decreased with distance from the index house to 6.2% in houses 80–100 meters away (p<0.001). Significantly more *Ae. aegypti* were DENV-infectious (i.e., DENV-positive in head/thorax) in positive clusters (23/1755; 1.3%) than negative clusters (1/1548; 0.1%). In positive clusters, 8.2% of mosquitoes were DENV-infectious in index houses, 4.2% in other houses with DENV-infected children, and 0.4% in houses without infected children (p<0.001). The DENV infection rate in contacts was 47.4% in houses with infectious mosquitoes, 28.7% in other houses in the same cluster, and 10.8% in positive clusters without infectious mosquitoes (p<0.001). *Ae. aegypti* pupae and adult females were more numerous only in houses containing infectious mosquitoes.

**Conclusions/Significance:**

Human and mosquito infections are positively associated at the level of individual houses and neighboring residences. Certain houses with high transmission risk contribute disproportionately to DENV spread to neighboring houses. Small groups of houses with elevated transmission risk are consistent with over-dispersion of transmission (i.e., at a given point in time, people/mosquitoes from a small portion of houses are responsible for the majority of transmission).

## Author Summary

Dengue is the leading cause of mosquito-borne viral infections globally. An improved understanding of the spatial and temporal distribution of dengue virus (DENV) transmission between humans and the principal vector, *Aedes aegypti*, can enhance prevention programs. Human DENV infection is known to occur at very fine spatiotemporal scales. We sought to link and quantify human DENV infections with infectious mosquitoes at these fine scales by conducting geographic cluster investigations around febrile children with and without DENV infection. We found that DENV infection in children was positively associated with houses in which infectious mosquitoes were captured. These houses also had more *Ae.* aegypti pupae and adult female mosquitoes than neighboring houses. However, the neighboring houses still had elevated rates of human DENV infection. Our results indicate that certain houses with high risk of DENV transmission contribute disproportionately to DENV amplification and spread to surrounding houses. At a given point in time, people and mosquitoes from a small portion of houses are responsible for the majority of DENV transmission.

## Introduction

Dengue is the most widespread mosquito-borne viral disease with 3.6 billion people at risk of infection world-wide each year [1]. *Aedes aegypti* is the principal mosquito vector of dengue virus (DENV). Indirect transmission occurs by horizontal transfer of virus between humans and female *Ae. aegypti* [2]. A key component of understanding DENV transmission dynamics is to understand the spatial and temporal scale at which human-mosquito encounters and virus transmission occur. DENV infection in humans has been shown to have substantial spatial and temporal variation at relatively small scales. Cohort studies in rural Thailand, where dengue is hyperendemic, indicate that dengue epidemiology and clinical presentation can differ dramatically between children in close geographic and temporal proximity at the level of a school and village [3]–[5]. Clusters of human DENV infections have also been detected in and around individual households [6]–[8]. Much of this fine scale spatiotemporal heterogeneity has been thought to be due, at least in part, to the behavior of the mosquito vector. Flight patterns and feeding behavior of the female *Ae. aegypti*, which have been studied extensively, indicate that this is a relatively sedentary species that feeds frequently and almost exclusively on human blood [9]–[15]. Much less is known about the interactions between humans and *Ae. aegypti* in natural settings that result in DENV transmission.

Results from our combined longitudinal cohort and geographic cluster study in Kamphaeng Phet, Thailand are consistent with focal DENV transmission occurring at a fine scale [5], [6]. Within 100 meters of a house with a DENV-infected child (as detected by school absence-based surveillance), the likelihood of another house with a DENV-infected child decreased with increasing distance from the original infected child's house. In the current report, we present additional data from the geographic cluster component of our larger cohort/cluster study that more specifically defines the dimensions of local transmission and quantifies the factors that support it. We detected a positive association between DENV infection in children and female *Ae. aegypti* at fine geographic and temporal scales. Our results add new details to the understanding of focal DENV transmission that can be used to further inform dengue surveillance and prevention strategies, and provide currently missing data for the construction, parameterization and validation of mathematical and simulation models of DENV transmission and control.

## Methods

### Ethics Statement

The study protocol was approved by the Institutional Review Boards of the Thai Ministry of Public Health (MOPH), Walter Reed Army Institute of Research (WRAIR), University of Massachusetts Medical School (UMMS), University of California at Davis (UCD) and San Diego State University (SDSU). Written informed consent was obtained from the parents of study participants and assent was obtained from study participants older than seven years.

### Study Location and Population

Our study methodology was previously described [5], [6]. Briefly, the geographic cluster study presented here was part of a larger combined longitudinal cohort and geographic cluster study conducted from 2004 to 2007 among children living in Muang district, Kamphaeng Phet province in north-central Thailand. Children came from 11 schools and 32 villages consisting of >8,445 houses. Demographics of house residents and house spatial coordinates were geo-coded into a Geographic Information System (GIS) database (MapInfo [2000] version 6·0; MapInfo Corporation).

### Geographic Cluster Investigations

Geographic cluster investigations were initiated by “index” cases selected from a longitudinal cohort of approximately 2000 primary school children. Active school absence-based surveillance was used to detect symptomatic DENV infection in the cohort from June to November of each study year [5]. Cohort children who were DENV-positive by semi-nested reverse transcriptase polymerase chain reaction (RT-PCR) [16] from an acute blood sample drawn within three days of illness onset served as an “index” case to initiate a positive cluster investigation around the index case house. Cohort children who were dengue PCR-negative from an acute illness blood sample served as an “index” case for a negative (i.e., control) cluster investigation. In each geographic cluster, ten to 25 child contacts aged six months to 15 years living within 100 meters of the index case were enrolled regardless of clinical status. The child contacts were evaluated at days 0 (i.e., the same day as cluster initiation), 5, 10, and 15 by temperature measurement and symptom questionnaire. Blood samples were collected on days 0 and 15. Paired day 0 and 15 blood samples from child contacts were tested by both dengue PCR and an in-house dengue/Japanese encephalitis IgM/IgG capture EIA [17]. Dengue EIA-positive results were categorized as “recent dengue” (RD) if IgM was negative but IgG was positive with a declining titer between days 0 and 15 [18], “enrollment seroconversion” (ES) if IgM was positive on both days 0 and 15, and “post-enrollment seroconversion” (PES) if IgM was negative on day 0 but positive on day 15. Based on estimated antibody kinetics and human incubation period [19], [20], the approximate interval between infection and day 0 blood collection for RD infections was thought to be about 3 weeks or more, ES infections up to about 2 weeks, and PES infections to be several days. Day 15 PCR-positive infections were thought to have occurred at or soon after cluster initiation.

### Entomological Procedures

On day 1 of each cluster investigation, adult *Ae. aegypti* were collected using backpack aspirators from inside and within the immediate vicinity of each house within a cluster. *Ae. aegypti* larvae and pupae were collected from water-holding containers [21]. After mosquito collections were completed, a pyrethrin mixture insecticide spray (BP-300: Pyronyl oil concentrate OR-3610A, Prentiss Inc.) was applied by ultralow volume aerosol inside and around each house to kill adult mosquitoes with the intention of terminating local DENV transmission [22]. Temephos was applied to artificial water holding containers to kill immature mosquitoes. On day 7, the Thai Ministry of Public Health (MOPH) sprayed deltamethrin or permethrin 10% in and around each house in a cluster according to their standard procedures.

Female *Ae. aegypti* were processed so that individual, serotype-specific rates for DENV-infectious mosquitoes could be detected. Mosquito abdomens were removed so that only those females that had virus particles in the head or thorax (i.e., disseminated infections with presumably infective salivary glands) were identified. Individual heads and thoraces were stored at −70°C in the field laboratory and transported weekly on dry ice to the Armed Forces Research Institute of Medical Sciences (AFRIMS) laboratory in Bangkok. At the AFRIMS laboratory, heads and thoraces of individual mosquitoes were ground and suspended in 100 µL of RPMI with 1% L-glutamine and 10% heat-inactivated FBS. Ten mosquito suspensions were pooled by combining 14 µL from each individual suspension. Pools were then tested by dengue PCR and each individual sample from a PCR-positive pool was tested by using 14 µL of the individual suspension diluted times ten [23], [24].

### Statistical Analysis

Data were analyzed using SPSS (SPSS for Windows version 19). Demographic, environmental and entomological parameters were analyzed at the cluster and house levels. Student's *t*-test or analysis of variance (ANOVA) was used to determine differences in continuous variables including distances between houses. Chi-square or Fisher's exact test was used for proportions. A mixed-effects logistic regression model was used to analyze the probability of infection of cluster contacts, while accounting for the nesting of observations within cluster investigations.

## Results

### DENV Infections in Child Contacts in Geographic Clusters

Of 805 child contacts enrolled in 50 positive cluster investigations, 129 (16.0%) had evidence of DENV infection; 119 (14.8%) were dengue EIA-positive on day 0 and/or 15 of which 40 were PCR-positive on day 0, and an additional 10 (1.2%) were DENV-positive only by PCR on day 15. In comparison, nine (1.1%) of 794 enrolled child contacts in 53 negative clusters had evidence of DENV infection; seven (0.9%) were dengue EIA-positive of which three were PCR-positive on day 0, and an additional two (0.3%) were DENV-positive by day 15 PCR alone [5].

**Figure 1:**
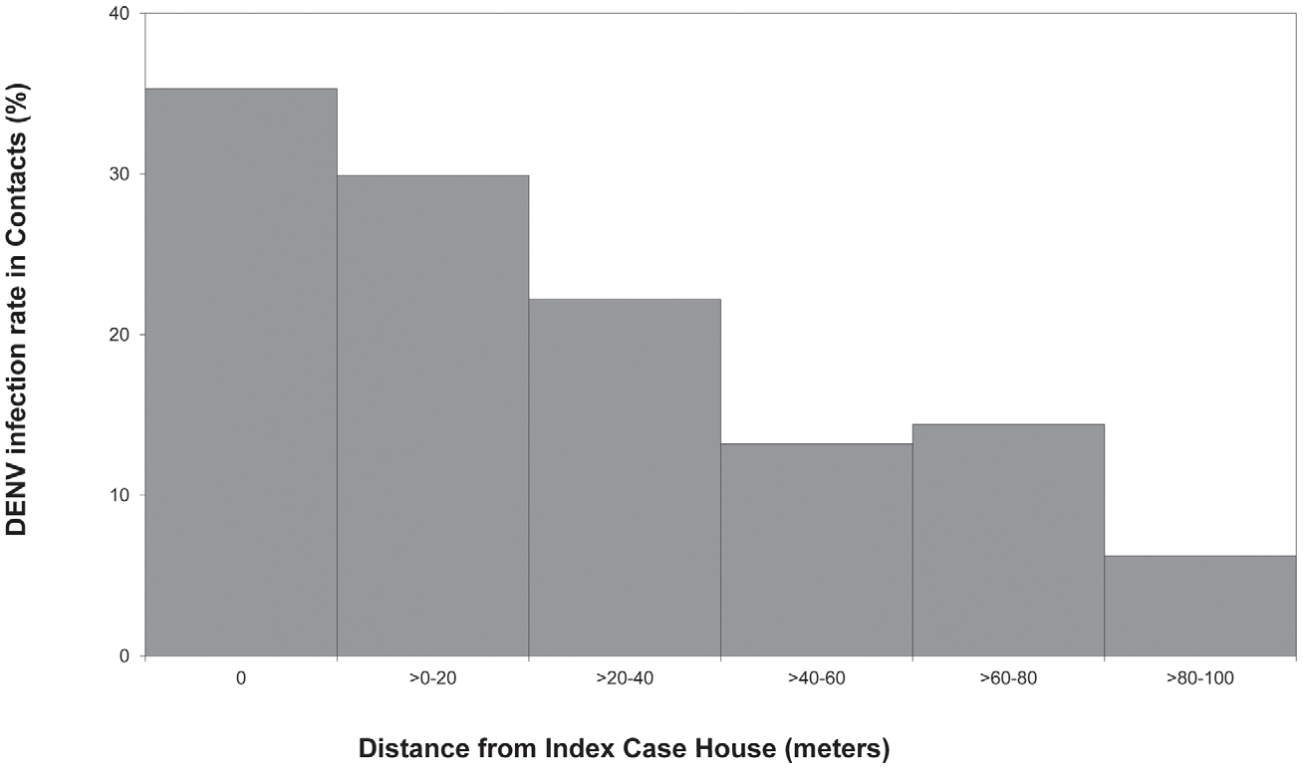
Focal aggregation of DENV-infected child contacts in positive clusters. Relationship between DENV infection rate and distance from index case house was significant (Chi square, p<0.001). Distance from index case house 0 m: N = 18/51; >0–20 m: N = 20/67; >20–40 m: N = 28/126; >40–60 m: 23/174; >60–80 m: 28/194; >80–100 m: 12/193 (N = Infected Contacts/Enrolled Contacts).

Within positive clusters, the percentage of enrolled contacts that were dengue EIA-positive varied significantly according to distance from the index case house. The DENV infection rate among contacts from the same house as a positive index case was 35.3%. If the child contact lived in a different house but within 20 meters of the index case house, the infection rate was 29.9%. The infection rate decreased with increasing distance from the index case house, down to 6.2% when the contact lived 80–100 meters away. The inverse relationship between DENV infection rate among contacts and distance from the index case house was significant (Chi square, p<0.001) (Figure 1). A mixed-effects logistic regression model confirmed that this association remained significant after controlling for age and gender (Table 1).

**Table 1:** Mixed-effects logistic regression analysis of the probability of DENV infections in child contacts in positive clusters.

Independent Variable	Coefficient	Standard Error	p-value
**Distance from Index Case House (m)**	−0.022	0.004	<0.001
**Age (yrs)**	0.006	0.028	0.823
**Female**	−0.165	0.224	0.460
**Intercept**	−0.982	0.390	0.012

**Figure 2:**
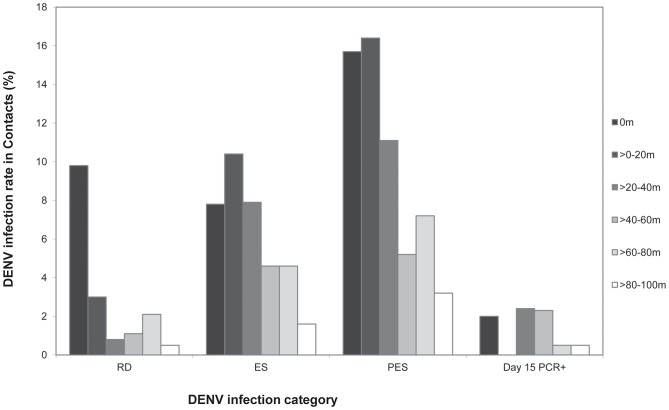
Focal aggregation of DENV-infected child contacts in positive clusters categorized by estimated time of infection. RD = recent dengue (N = 15); ES = enrollment seroconversion (N = 41); PES = post-enrollment seroconversion (N = 63); day 15 PCR-positive (N = 10).

Of the 119 dengue EIA-positive child contacts in the positive clusters, 15 (12.6%) were categorized as having RD infection, 41 (34.5%) as ES infection, and 63 (52.9%) as PES infection. RD, ES and PES infections in the positive clusters all tended to decrease as the distance from the index case house increased (Figure 2). DENV infections based solely on a day 15 PCR-positive result did not appear to decrease with increasing distance from the index case house; however, the number of these cases was low (Figure 2).

### DENV Infections in Female *Ae. aegypti* in Geographic Clusters

Twenty-three (1.3%) of 1755 female *Ae. aegypti* were dengue PCR-positive in positive clusters, while one (0.1%) of 1548 was PCR-positive in negative clusters (p<0.001). Considering only those houses with index cases or enrolled child contacts, all 19 DENV-infectious female *Ae. aegypti* collected from these houses were in positive clusters. These 19 mosquitoes came from 16 different houses in 14 different positive clusters (Table 2). All four DENV serotypes were represented.

**Table 2:** Description of houses with DENV-infectious female *Ae.* aegypti.

House	Cluster month/year	Index case house (Y/N)	Female *Ae. aegypti* collected	Infectious female *Ae. aegypti*	*Ae. aegypti* pupae	Human adults	Human children	Enrolled child contacts	DENV infection in enrolled contacts	DENV serotype of infectious mosquito	DENV serotype of index case in same cluster	DENV serotype of infected child contact
1	Jun 04	N	7	1	164	3	2	2	1	DENV-4	DENV-4	ND
2	Jun 04	Y	3	1	5	2	3	2	1	DENV-4	DENV-4	ND
3	Sep 04	N	8	2	129	4	1	1	1	DENV-4, DENV-4	DENV-4	ND
4	Nov 05	N	17	1	57	1	1	0	0	DENV-3	DENV-3	-
5	Nov 05	Y	2	1	6	2	1	1	0	DENV-3	DENV-3	-
6	Jun 06	Y	9	1	1	6	2	1	0	DENV-1	DENV-1	-
7	Jun 06	Y	7	1	2	2	1	0	0	DENV-4	DENV-4	-
8	Jun 06	N	1	1	3	3	2	2	1	DENV-4	DENV-4	DENV-4
9	Aug 06	N	5	1	3	3	3	2	2	DENV-1	DENV-1	ND, ND
10	Aug 06	Y	2	1	5	4	2	1	1	DENV-4	DENV-4	ND
11	Oct 06	Y	2	1	0	3	3	2	1	DENV-1	DENV-1	DENV-1
12	Nov 06	Y	3	1	2	4	1	0	0	DENV-2	DENV-2	-
13	Jun 07	N	4	1	0	2	3	3	0	DENV-1	DENV-1	-
14	Jun 07	N	3	1	1	3	1	1	0	DENV-1	DENV-1	-
15	Jul 07	Y	15	3	0	3	1	0	0	DENV-1, DENV-1, DENV-1	DENV-1	-
16	Jul 07	N	4	1	0	2	1	1	1	DENV-2	DENV-2	ND
**Mean per house (s.d.)**	**-**	**-**	**5.75 (4.65)**	**1.19 (0.54)**	**23.6 (50.3)**	**2.94 (1.18)**	**1.75 (0.86)**	**1.19 (0.91)**	**0.56 (0.63)**	-	-	-

Footnote: Includes only houses with index cases or enrolled child contacts. ND = not detected by PCR but positive by EIA.

Houses with DENV-infectious mosquitoes had significantly more *Ae. aegypti* pupae and total female *Ae. aegypti* mosquitoes than houses without infectious mosquitoes (Table 3). Two of the houses with infectious mosquitoes (houses 1 and 3) contained the largest and second largest number (164 and 129) of *Ae. aegypti* pupae collected from any house in the entire study (Table 2).

**Table 3:** Selected entomological indices and DENV infection rates in houses with and without infectious mosquitoes.

	Positive Clusters				Houses in Negative Clusters	Difference p-value between positive and negative cluster houses
	Houses with Infectious Mosquitoes (19 Infectious Mosquitoes)	Houses in same cluster as Infectious Mosquitoes but with no Infectious Mosquitoes	Houses in clusters with no Infectious Mosquitoes	Difference p-value		
**Houses, N**	16	158	324	-	480	-
**Female ** ***Ae. aegypti*** **, N per person (s.d.)**	1.57 (2.05)	0.49 (1.00)	0.45 (0.81)	<0.001	0.31 (0.49)	<0.001
***Ae. aegypti*** ** pupae, N per person (s.d.)**	5.84 (11.59)	1.86 (3.76)	1.22 (2.56)	<0.001	1.39 (2.99)	0.383
**Human adults, N per house (s.d.)**	2.94 (1.18)	2.92 (1.31)	2.70 (1.44)	0.239	2.74 (1.25)	0.607
**Human children, N per house (s.d.)**	1.75 (0.86)	1.68 (0.88)	1.77 (1.04)	0.598	1.82 (1.10)	0.272
**DENV-infected child contacts/Enrolled child contacts (%)**	9/19 (47.4)	56/195 (28.7)	64/591 (10.8)	<0.001	9/794 (1.1)	<0.001

Footnote: Only houses with index cases or enrolled child contacts were included. Person refers to adults and children. P-values are for one-way ANOVA comparisons for means and Fisher's exact test for proportion of infected contacts.

### Relationship between DENV Infection in Children and Female *Ae. aegypti*

There were 17 DENV infections in children from the 16 houses with DENV-infectious mosquitoes; 9 DENV infections were in child contacts and 8 were in index cases. Within the houses with infectious mosquitoes, the serotype, when available, of DENV in infected children was identical to that in the infectious mosquito from the same house (Table 2).

DENV infection in children was positively associated with the presence of DENV-infectious mosquitoes in the house. The DENV infection rate among child contacts in houses with infectious mosquitoes was 47.4% compared to 28.7% in houses from the same cluster but without infectious mosquitoes, and 10.8% in houses from other positive clusters (Fisher's exact, p<0.001; Table 3). Conversely, the DENV infection rate among female *Ae. aegypti* from houses with a positive child index case was 8.2% (Table 4). Excluding index case houses, the rate of infectious mosquitoes from positive cluster houses with a DENV-infected child was 4.2%. This rate was only 0.4% when no child was infected in a positive cluster house (Fisher's exact, p<0.001).

**Table 4:** Rates of DENV-infectious *Ae. aegypti* in houses with and without DENV-infected children in positive clusters.

	Houses with index case (N = 50)	Houses with DENV-infected contacts, not including houses with index case (N = 86)	Houses with no DENV-infected contacts (N = 395)	Difference p-value
**DENV-infectious ** ***Ae. aegypti*** **/Collected female ** ***Ae. aegypti*** ** (%)**	10/122 (8.2)	6/142 (4.2)	3/735 (0.4)	<0.001
**Human adults, N per house (s.d.)**	2.70 (1.18)	2.83 (1.30)	2.78 (1.44)	0.879
**Human children, N per house (s.d.)**	2.14 (1.51)	1.90 (1.01)	1.65 (0.88)	0.001
**Female ** ***Ae. aegypti*** ** per person (s.d.)**	0.65 (1.35)	0.46 (1.19)	0.49 (0.83)	0.492
**DENV-infectious ** ***Ae. aegypti*** ** per person (s.d.)**	0.049 (0.140)	0.016 (0.067)	0.002 (0.024)	<0.001

Footnote: Person refers to adults and children. Only houses with index cases or enrolled child contacts were included. P-values are for one-way ANOVA comparisons for means and Fisher's exact test for proportions of infectious mosquitoes.

**Figure 3:**
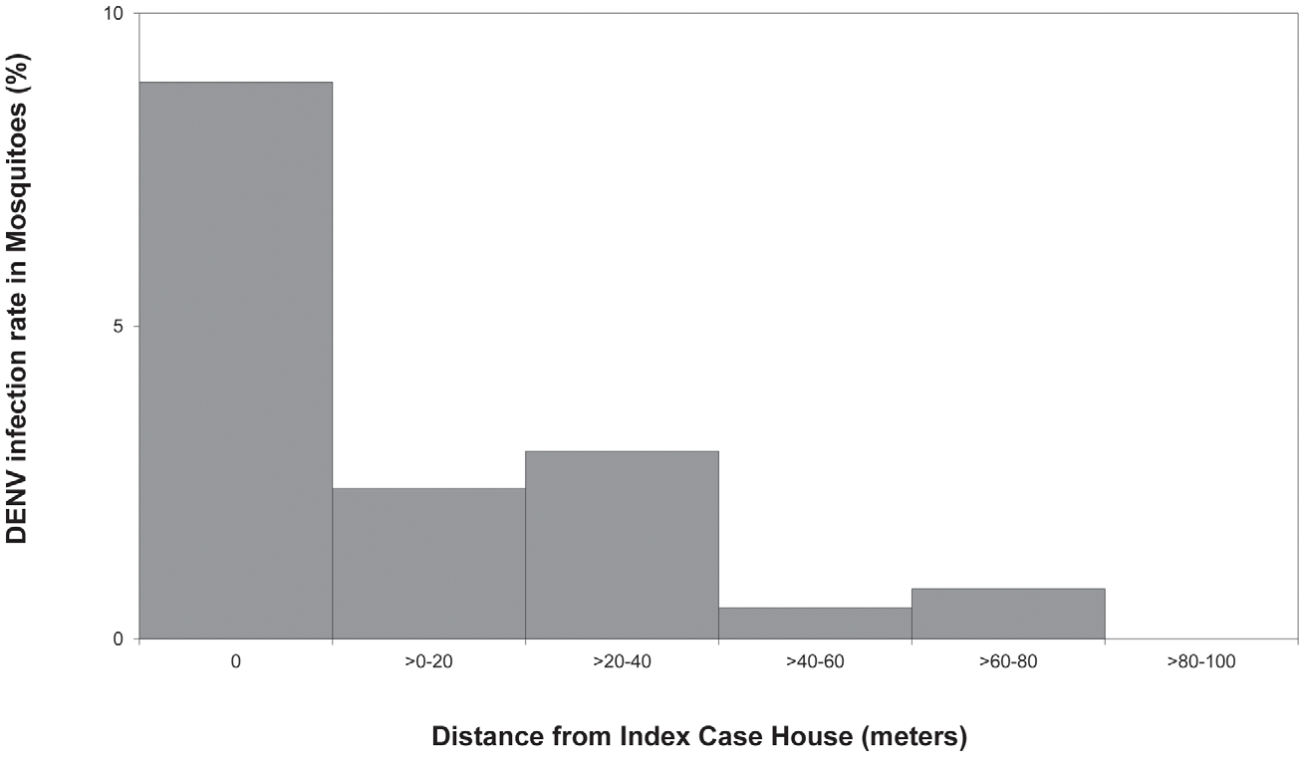
Focal aggregation of DENV-infectious mosquitoes in positive clusters. Relationship between proportion of female *Ae. aegypti* that were DENV-infectious and distance from index case house was significant (Fisher's exact, p<0.001). Distance from index case house 0 m: N = 10/112; >0–20 m: N = 2/85; >20–40 m: N = 4/138; >40–60 m: 1/204; >60–80 m: 2/243; >80–100 m: 0/206 (N = Infectious mosquitoes/Collected mosquitoes).

Within the 100-meter radius of positive clusters, almost all DENV-infectious *Ae. aegypti* were collected from index case houses or from houses within 40 meters of the index case house (Figure 3). This negative correlation of infectious mosquitoes with distance from the index case house was most likely due to the positive association between houses containing infected children and infectious mosquitoes.

**Figure 4:**
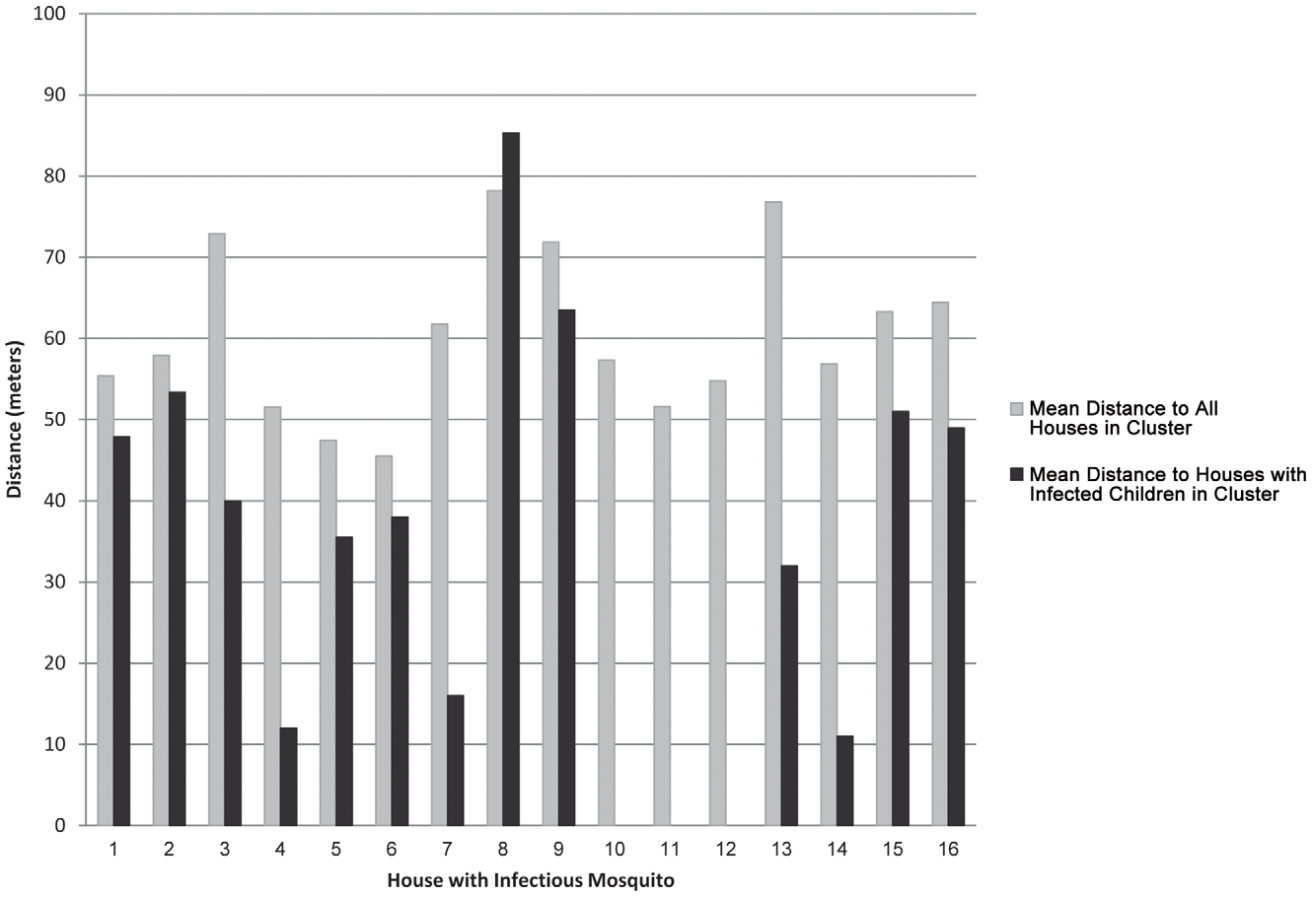
Mean distance of houses with DENV-infectious mosquitoes to other houses in the same cluster.

Within an individual positive cluster, houses with DENV-infectious mosquitoes tended to be closer to houses containing DENV-infected children than to all houses. Figure 4 shows the mean distance between each of the 16 houses with infectious mosquitoes and other houses in their respective clusters. In three houses (#10, 11 and 12), the only infected children in the cluster were in the houses with the infectious mosquito(es). Of the 13 possible comparisons, 12 houses with infectious mosquitoes were closer to houses with infected children than to all houses within the cluster. On average, houses with infected children were closer to houses with infectious mosquitoes than to houses with no infectious mosquitoes within their respective positive clusters (p = 0.028).

## Discussion

This study demonstrates a positive association between DENV-infectious *Ae. aegypti* and DENV-infected children living in the same and neighboring houses. Spatiotemporal clustering of DENV infection in children and mosquitoes was detected at a fine scale, consistent with focal aggregation well within a 100-meter radius area. Houses with infectious mosquitoes had an especially high risk (47.4%) of human DENV infection along with elevated measurements of mosquito density; neighboring houses also had elevated risk of human infection. Our results are consistent with the notion that houses with high DENV transmission risk contribute disproportionately to virus amplification and spread. Infections followed a pattern of over-dispersion, which has been reported for other infectious diseases to include indirectly transmitted, mosquito-borne infections [25]–[28]. At a given point in time, people and mosquitoes in a relatively small portion of houses were responsible for the majority of DENV transmission.

Our results are the first to demonstrate a direct relationship between DENV infection in humans and mosquitoes at very fine spatiotemporal scales in the natural setting. Other researchers have reported heterogeneity of human DENV infection across space and time [4], [6], [8], [29]. Many entomological studies have shown the limited flight range and preferential and frequent human feeding behavior of *Ae. aegypti* that would be expected to enhance DENV transmission [11], [13]–[15], [22], [30]. Prior studies of DENV infections in mosquitoes tended to focus on mosquitoes collected in or around houses of people with dengue-like illness [31], [32]; and when these studies were done across communities, infected mosquitoes were not explicitly linked to human infection [33]. Perhaps because of the difficulty in collecting adult *Ae. aegypti*, there has been relatively little research done on mosquito DENV infections in relation to human infection dynamics. Our study expands on this picture by showing that human and mosquito infections are positively associated with each other at small geographic and temporal scales. The strongest association was at the level of the individual house.

We did not directly evaluate the role of human adults in DENV transmission. It is possible that spatiotemporal dynamics of DENV transmission is different in adults and children, perhaps due to age-specific differences in existing immunity, the rate at which they are bitten [34], or in their movement patterns and exposure to daytime-biting *Ae. aegypti* [35]. We would not expect our overall conclusions to change, however, because both adults and children would have contributed to our findings whether or not adults were separately evaluated.

Fine scale spatial aggregation of DENV transmission may persist for three weeks or longer. Given the estimated time of infection of RD, ES and PES infections and because all of these categories of infection appeared to show focal aggregation within the ≤100-meter radius of the clusters, the spatial pattern we detected could have been present for greater than three weeks. This pattern, which is similar to what was observed for DENV-infected *Ae. aegypti* in households in Mexico [12], may have persisted for a longer period if not truncated by the vector control interventions instituted on day 1 (by the study team) and day 7 (by the MOPH) of the cluster investigations. The lack of focal aggregation among day 15 PCR-positive child contacts supports this notion, although the small number of those infections may have been insufficient for a meaningful analysis.

Our testing method favored identification of PCR-positive mosquitoes that were infectious. The DENV incubation period in mosquitoes from the time that they imbibe an infectious blood meal to the time they become infectious (i.e., extrinsic incubation period) typically lasts for 10–14 days under environmental conditions like those in Kamphaeng Phet, Thailand [2], [36]. This implies that DENV-infectious mosquitoes in our study fed on an infected human considerably earlier than the time of cluster initiation and, thus, the transmission chain in houses with infectious mosquitoes had been taking place for some time before the “index” case was detected and the cluster investigation initiated. Consequently, as with infected children in the clusters, focal aggregation of infectious mosquitoes within the clusters may have been going on for two weeks or longer prior to initiation of each cluster investigation. So although “index” cases were used to initiate cluster investigations, they were not necessarily the first infection to occur within the cluster. Again, because vector control measures were instituted on day 1 and 7 and no further entomological collections were performed afterwards, we were not able to determine how long the focal pattern of DENV infection in mosquitoes would have persisted. We speculate that the duration of these focal areas of higher risk is limited more by the availability of susceptible humans than by susceptible mosquitoes. Future studies could investigate the required duration of interventions, which may need to be continued for one month or more.

Significantly more *Ae. aegypti* pupae and adult females were collected from houses containing infectious mosquitoes than from those without. In addition, the risk of DENV infection in children was high in houses with infectious mosquitoes and, notably, remained elevated in neighboring houses. The higher entomological indices, however, were detected only in houses that actually contained infectious mosquitoes. These findings indicate that certain individual houses with high DENV transmission risk may disproportionately contribute to virus transmission within neighboring houses, likely due to local human and mosquito movement. Our study did not specifically evaluate when these elevated entomological measurements began or how long they persisted. They could have been present for some time prior to detection. Therefore, even in clusters with high DENV transmission, there may be individual houses that are responsible for the bulk of the transmission risk. Dengue management interventions that fail to include these individual, high-risk houses may have less impact than expected on reducing overall DENV spread. Similarly, surveillance programs that average measurements or indices of risk over a large area may fail to detect individual high-risk houses that disproportionately contribute to persistence and expansion of local transmission [25].

Fine scale spatiotemporal clustering of human-mosquito DENV transmission supports the hypothesis that DENV spread to more distant locations is driven by human movement [35]. Whether DENV is successfully transmitted at those distant locations is likely related to a suite of factors including susceptibility of the local human population, mosquito vector density and infection status, vector competence, degree of human-vector contact, and intrinsic virus factors. Locations with high levels of human movement and potential for high interaction between people and mosquitoes merit additional investigation. These components of transmission may need to be factored into dengue surveillance and control efforts more than is currently being done [37].

Results from our study have implications for strategies to prevent DENV transmission. Transmission models that address DENV spread and the impact of vaccines alone or in combination with vector control need to account for the spatiotemporal scale and dynamics of DENV transmission. Depending on the questions being asked, these models and the interpretation of surveillance data that feed into them will need to account for the presence of high-risk hotspots of human-vector virus exchange that have a high impact on DENV spread to surrounding areas [27], [28], [38]. These efforts should be integrated into an overall multifaceted strategy that takes into account DENV spread by movement of viremic humans among focal areas of concentrated, high levels of transmission.

## Supporting Information

Checklist S1 **STROBE checklist.**
(DOC)
